# Stroma gene signature predicts responsiveness to chemotherapy in pancreatic ductal adenocarcinoma patient‐derived xenograft models

**DOI:** 10.1002/1878-0261.13816

**Published:** 2025-02-04

**Authors:** Alessia Anastasia, Laura Formenti, Paola Ostano, Lucia Minoli, Andrea Resovi, Lavinia Morosi, Claudia Fioravanti, Edoardo Micotti, Cristina Matteo, Eugenio Scanziani, Giovanna Chiorino, Raffaella Giavazzi, Carmen Ghilardi, Dorina Belotti

**Affiliations:** ^1^ Department of Oncology Istituto di Ricerche Farmacologiche Mario Negri IRCCS Bergamo and Milan Italy; ^2^ Lab of Cancer Genomics Fondazione “Edo ed Elvo Tempia” Biella Italy; ^3^ Department of Veterinary Medicine and Animal Sciences (DIVAS) University of Milan (Unimi) Lodi Italy; ^4^ Department of Neuroscience Istituto di Ricerche Farmacologiche Mario Negri IRCCS Milan Italy; ^5^ Mouse and Animal Pathology Laboratory (MAPLab), Fondazione Unimi Milan Italy; ^6^ Present address: IRCCS Humanitas Research Hospital Rozzano Italy

**Keywords:** pancreatic cancer, patient‐derived xenografts, stroma signature, treatment response

## Abstract

Despite many efforts to understand the molecular mechanisms of pancreatic ductal adenocarcinoma (PDAC) treatment resistance, there is still no reliable method for selecting patients who could benefit from standard pharmacological treatment. Here, four PDAC patient‐derived xenografts (PDAC‐PDXs) with different responses to gemcitabine plus nab‐paclitaxel (nanoparticle albumin‐bound paclitaxel) were studied to dissect the contribution of both tumor and host microenvironment to treatment response. PDAC‐PDXs transplanted into the pancreas of immunodeficient mice retained the main genetic and histopathological characteristics of the original human tumors, including invasiveness and desmoplastic reaction. Response to chemotherapy was associated with a specific 294 stroma gene signature and was not due to the intrinsic responsiveness of tumor cells or differences in drug delivery. Human dataset analysis validated the expression of the 294 stroma gene signature in PDAC clinical samples, confirming PDAC‐PDXs as a useful tool to study the biology of tumor–host interactions and to test drug efficacy. In summary, we identified a stroma gene signature that differentiates PDAC‐PDXs that are responsive to gemcitabine plus Nab‐paclitaxel treatment from those that are not, confirming the active role of the tumor microenvironment in the drug response.

## Graphical abstract

Pancreatic ductal adenocarcinoma patient‐derived xenografts (PDAC‐PDXs) engrafted orthotopically in the pancreas of immunodeficient mice retain the main genetic and histopathological characteristics of the original human tumors. A 294 stroma gene signature differentiates between PDAC‐PDXs that are responsive to gemcitabine plus nab‐paclitaxel versus those that are nonresponsive, suggesting an active role of the tumor microenvironment in drug response.

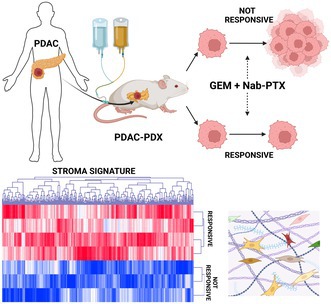

**Abbreviations**
CPS: pancreatitis fibrotic tissue
Crem‐PTX: cremophor EL‐paclitaxel
Gem: gemcitabine
GEO: Gene Expression Omnibus
GO: Gene Ontology
H&E: hematoxylin and eosin
HDE: human dose equivalent
HPLC: high‐performance liquid chromatography
MRI: magnetic resonance imaging
MSI: mass spectrometry imaging
Nab‐PTX: nanoparticle albumin‐bound paclitaxel
NE: normal ducts of pancreas
PDAC: pancreatic ductal adenocarcinoma
PDAC‐PDXs: PDAC patient‐derived xenografts
ROIs: regions of interest
SCID: severe combined immunodeficiency
TE: tumor epithelium
TGCA: the Cancer Genome Atlas
TS: tumor stroma

## 1 Introduction

Pancreatic ductal adenocarcinoma (PDAC), one of the most aggressive tumors, is the sixth leading cause of global cancer deaths [1]. Less than 9% of PDAC patients survive 5 years from diagnosis [2, 3]. The poor prognosis is due to late diagnosis and lack of effective therapies. To date, surgery is the only potentially curative treatment. However, approximately 85% of the patients present with advanced unresectable disease and receive chemotherapy in a neo‐adjuvant or adjuvant setting. Efforts have been made to define the best chemotherapeutic regimen, but in most cases, there are only slight improvements in survival, and even after chemotherapy, the majority of patients experienced recurrences [2].

Gemcitabine alone or combined with nab‐paclitaxel remains the standard of care for PDAC, while FOLFIRINOX is generally chosen for patients with a good performance status, because of its high toxicity [4]. Molecularly targeted drugs and immunotherapy offer interesting new prospects, which are the objective of intensive studies also in combination with standard treatments [5]. However, unlike a number of solid malignancies that successfully respond to immunotherapy, PDAC orchestrates several mechanisms of immune‐escape that limit the efficacy of vaccines and immune checkpoint inhibitors [6]. Similarly, treatments with angiogenesis‐ or PARP inhibitors gave various different effects with limited efficacy in terms of tumor response and patient survival [7, 8].

Heterogeneity in treatment response and failure of numerous clinical trials in PDAC are partly due to the different molecular tumor subtypes [9] though also partly to the extensive tumor stroma (including extracellular matrix and a wide range of cells like fibroblasts, cancer‐associated fibroblasts, endothelial cells, pericytes, adipocytes, myeloid and lymphoid immune cells) that interacts with the tumor cells, influencing tumor progression and drug response [10, 11]. Faced with this molecular diversity, it is challenging to find effective therapeutic approaches with unselected patient recruitment in clinical trials.

In the last few years, RNA and proteomic signatures have cast light on the molecular heterogeneity of PDAC [12] and different subtypes have been associated with patient prognosis and response to treatments [13, 14]. However, given the prominent role of the tumor microenvironment in PDAC, its better characterization should give further clues for effective therapeutic strategies.

In this study, four patient‐derived xenografts (PDXs) successfully engrafted orthotopically in the pancreas of immunodeficient mice were molecularly and pharmacologically characterized [15]. Although all four belonged to the ‘classical PDAC subtype’ [13], they responded differently to gemcitabine plus Nab‐paclitaxel. Pharmacokinetic and mass spectrometry imaging (MSI) analysis showed that the differences were not caused by altered drug distribution linked to diverse amounts of stroma in the tumors. Using transcriptomic analysis, we were able to show that the response to gemcitabine plus Nab‐paclitaxel was associated with a specific stroma PDX signature.

## 2 Materials and methods

### 2.1 Animals

Six‐ to eight‐week‐old female C.B‐17 severe combined immunodeficiency (SCID) mice were obtained from Envigo, S. Pietro al Natisone, Italy. Mice were maintained under specific pathogen‐free conditions, housed in isolated vented cages, and handled using aseptic procedures. Procedures involving animals and their care were conducted in conformity with institutional guidelines that comply with national and international laws and policies (Legislative Decree 26, March 2014) (Directive 2010/63/EU adopted on 22 September 2010), in line with guidelines for the welfare and use of animals in cancer research [16]. Animal studies were approved by the Mario Negri Institute Animal Care and Use Committee and by Italian Ministerial decree (Authorization no. 85/2013‐B and no. 519/2021‐PR).

### 2.2 PDAC‐PDX models

PDAC‐PDXs were established as described previously [15]. The study methodologies were conformed to the standards set by the Declaration of Helsinki and approved by Presidio Ospedaliero of Rho ethics committee (authorization no. 00341/2013/DG; 17 June 2013). The samples were collected with understanding and the written consent of each patient from 2013 to 2015.

PDAC‐PDXs were used at the third or fourth passage after the first engraftment from patients. Tumor fragments were split into 2 × 2 mm pieces and implanted orthotopically into the head of the pancreas or subcutaneously in the flank of C.B‐17 SCID mice, as described in detail below. The orthotopic tumor growth was evaluated by weekly abdominal palpation and magnetic resonance imaging (MRI). The subcutaneous tumor growth was measured with a Vernier caliper, and tumor volume was calculated as [(length × width^2^)/2 = mm^3^].

PDAC‐PDXs growing orthotopically in the pancreas were used for histological analysis, mutation screening, gene expression, and drug testing as described below.

### 2.3 Histopathology and immunohistochemistry

Pancreatic tumor masses were collected at euthanasia, fixed in 10% neutral buffered formalin, and paraffin‐embedded for histopathological analyses; 4 μm serial sections were routinely stained with hematoxylin and eosin (H&E), Sirius Red, and Alcian Blue [17]. Immunohistochemistry was done using the following primary antibodies: anti‐human leukocyte antigen (ab52922; Abcam, Cambridge, UK, specific for cells of human origin), anti‐Vimentin EPR3776 (ab92547; Abcam, specific for mesenchymal cells of both human and murine origin), anti‐Vimentin sp20 (Thermo Fisher Scientific, Waltham, MA, USA, specific for mesenchymal cells of human origin), anti‐P16 INK4A (E‐AB‐13142; Elabscience), and anti‐SMAD4 (E‐AB‐16957; Elabscience, Houston, TX, USA).

Tumor desmoplasia was quantified by digital image analysis on Sirius Red‐stained slides. Quantification was done on the whole digitalized section with orbit.bio software (http://www.orbit.bio), and results were expressed as the percentage of the positive area in the whole tumor.

### 2.4 Mutation screening

*KRAS* (exon 2), *TP53* (exons 5–9), *CDKN2A* (exons 1–4), and *SMAD4* (entire coding sequence) were sequenced to assess their mutational status in PDAC‐PDXs.

Genomic DNA (for *KRAS*, *TP53*, and *CDKN2A* analysis), extracted using the Maxwell® 16 DNA Purification kits (Promega, Madison, WI, USA), and total RNA (for *SMAD4* analysis), isolated from PDAC‐PDX using miRNeasy Mini Kit (Qiagen, Hilden,Germany), were quantified by NanoDrop 1000 Spectrophotomoter (Thermo Fisher Scientific). To obtain cDNA, 500 ng total RNA was reverse transcribed with 2 pmols of SMAD4 reverse primer, 0.7 mm dNTPs mix, and 200 U SuperScript™ II RT (Thermo Fisher Scientific) at 42 °C for 50 min.

Specific amplicons were PCR amplified in 50 μL reaction mix, containing 50 ng DNA or 2 μL cDNA, 200 μm dNTP solution, 200 nm specific primers, and 1 U FastStart Taq Polymerase (Roche, Basel, Switzerland) using a 2720 Thermal Cycler (Applied Biosystems, Foster City, CA, USA). Primer pairs were designed using primer‐3 software (https://primer3.ut.ee/). Cycling conditions were as follows: 35 cycles of denaturation at 94 °C for 15 s, annealing at 60 °C for 30 s, and extension at 72 °C for 45 s. Primer sequences are listed in Table S1. PCR products were purified using Illustra GFX™ PCR DNA and Gel Band Purification Kit (Cytiva, Wilmington DE, USA) and sequenced using Sanger's method by Eurofins (Vimodrone, Milan).

### 2.5 Gene expression microarray and analysis

RNA integrity and quality were checked using the Agilent 2100 Bioanalyzer (Agilent Technologies, Santa Clara, CA, USA). Only RNA with RNA integrity number (RIN) above 6 were used for transcriptomic analyses. Gene expression profiling was carried out using the one‐color labeling method. A total of 100 ng RNA were amplified, labeled with Cy3, and purified with columns. For every sample, 600 ng of labeled specimens was hybridized on Agilent Human Gene Expression v3 8x60K and Agilent Mouse Gene Expression v2 8x60K microarrays. After 17 h of hybridization, slides were washed and scanned using the Agilent Scanner version C (G2505C; Agilent Technologies). Images were analyzed using feature extraction software v10.7.3.1 (Agilent Technologies). Raw data were elaborated with bioconductor (www.bioconductor.org), using the limma (Linear Models for Microarray Analysis) R package. Background correction was done with the normexp method with an offset of 50, and quantile was used for between‐array normalization. The empirical Bayes method was used to compute a moderated *t*‐statistics for class comparison. Transcripts with an absolute fold change greater than 1.5 and a *P*‐value lower than 0.01 were considered as differentially expressed. Raw and processed data were deposited on the Gene Expression Omnibus (GEO) database (GSE234259).

### 2.6 External datasets

The following datasets were retrieved from GEO and ArrayExpress public repositories: GSE15471 [18], GSE16515 [19], GSE43795 [20], GSE32676 [21], E‐MEXP‐950, and E‐MEXP‐1121 [22]. Normalized data were downloaded and used for analysis.

Clinical and RNA‐sequencing profiles from pancreatic adenocarcinoma samples available in the TCGA database were also retrieved (https://gdac.broadinstitute.org/; accessed March 2023). Data on the TCGA profiles were analyzed with deseq2, and variance stabilizing transformation counts were used for subsequent analysis [23].

### 2.7 Cluster and functional analysis

mev version 4.9.0 (https://sourceforge.net/projects/mev‐tm4/files/mev‐tm4/MeV%204.9.0/) was used for unsupervised hierarchical clustering on the gene expression profiles of selected datasets. Over‐represented biological processes of the Gene Ontology (GO) and pathways were investigated with the functional annotation tool available in DAVID (https://david.ncifcrf.gov/).

### 2.8 Drug preparation and PDAC‐PDX treatment

Nab‐Paclitaxel (Nab‐PTX, Abraxane®; Celgene, Brystol Myers, Princeton, NJ, USA) was dissolved in saline and injected intravenously (i.v.) at a dose of 25 mg·kg^−1^ corresponding to 75 mg·m^−2^ Human Dose Equivalent (HDE). Paclitaxel (PTX; Indena S.p.A., Milano, Italy) was dissolved in 50% Cremophor EL (Sigma‐Aldrich, Taufkirchen, Germany) and 50% ethanol, further diluted with saline immediately before use, and then injected i.v. at a dose of 25 mg·kg^−1^ corresponding to 75 mg·m^−2^ HDE. Gemcitabine (Gem; Teva Italia, Milano, Italy) was dissolved in saline and injected i.v. at a dose of 150 mg·kg^−1^ corresponding to 450 mg·m^−2^ HDE. Drugs were given on Days 1 and 8 of each 21‐day cycle for four cycles, according to the patients' schedule.

#### 2.8.1 Orthotopic setting

Tumor fragments were implanted in the pancreas of C.B‐17 SCID female mice, and tumor growth was monitored by abdominal palpation. After 50–60 days, tumors were analyzed with MRI and randomized by tumor burden, arbitrarily assigning four to six mice per group to the different treatments. Tumor growth was subsequently monitored by MRI during and at the end of treatment (fourth cycle) and, whenever possible, 1 and 3 months after stopping the treatment. For each mouse, data were expressed as: % change = [(volume at time *t* − starting volume)/starting volume] × 100. According to the RECIST guideline [24], disease was considered progressive if the change in tumor volume was greater than 20%, stable if it was between ‐30% and 20%, and regressive if it was lower than ‐30%. Mice were euthanized at the first signs of distress caused by the tumor progression.

#### 2.8.2 Ectopic setting

Tumor fragments were implanted subcutis in C.B‐17 SCID mice, which were randomized and arbitrarily assigned to the different treatments when the tumor reached the size specified in each experiment (stratified randomization; four to five mice per group). To generate the tumor growth rate/curve, the mean tumor volume for each group was plotted against the time from start of treatment. Mice were euthanized when tumors reached 1000–1500 mm^3^, not exceeding 15% of the animal body weight.

### 2.9 Magnetic resonance imaging

Animals were anesthetized with isoflurane in O_2_ (100%), keeping the isoflurane from 1.0% to 2.0% throughout the experiments. Body temperature was kept at ~ 37 °C with a circulating warm water heating cradle. Imaging was done with a 7 T small animal Scanner (Bruker Biospec, Ettlingen, Germany). Two actively decoupled radio frequency coils were used: a 7.2 cm diameter volume coil used as the transmitter and a 2 × 2 array surface coil as the receiver. Respiratory frequency was monitored by a mechano‐sensor on the chest. All analyses were done in a range of respiratory activity from 50 to 90 breaths·min^−1^.

After a coronal scout SE image, a RARE T2‐weighted sequence was done to assess the intrapancreas tumor. To reduce image artifacts, a respiration trigger system was adopted. The images were obtained with a voxel size of 117 × 117 μm (matrix = 256 × 172 and Field of View = 3 × 2 cm) and slice thickness of 0.8–1.2 mm depending on the tumor volume: TR = 4000 ms, effective; TE = 36 ms, two averages.

The volumes of MRI images were measured using a Java‐based image processing program (image j) (https://imagej.net/ij/). Regions of interest (ROIs) were selected manually by a trained operator. Total tumor volume measurements were worked out by selecting all the slices with the presence of tumor. For each single tumor section, the total area was first determined and the volume was calculated by multiplying the area (mm^2^) by the section thickness (between 0.8 and 1.2 mm). Total volume was obtained by adding the volume of each tumor section.

### 2.10 Drug delivery study and MALDI mass spectrometry imaging analysis

Mice bearing PDAC‐PDX (*n* = 8–9; tumor weight approximately 500–800 mg) were treated with Nab‐PTX, 60 mg·kg^−1^. After 4 h, mice were deeply anesthetized with isoflurane and blood was collected by intracardiac puncture into heparinized tubes, centrifuged for 10 min at 4000 ***g*** at 4 °C and the plasma fraction was separated. Mice were then killed by cervical dislocation, and tumors and livers were excised and immediately frozen in dry‐ice. Paclitaxel concentrations were measured in all the biological specimens by high‐performance liquid chromatography (HPLC), as described previously [25].

Mass spectrometry imaging was used to analyze the distribution of PTX within the tumor. The PTX ion signal was analyzed by imagej software. ROIs, including the whole tumor section, were drawn and a threshold was established. The number of pixels exceeding the threshold was calculated and divided by the total number of pixels in the ROIs to obtain the percentage of pixels where the drug was present [26, 27].

### 2.11 Statistical analysis

Statistical analysis was done with graphpad prism version 6 (Graphpad, LaJolla, CA, USA). The differences in drug delivery and Sirius Red quantification were analyzed with an unpaired *t*‐test. *P*‐value < 0.05 was considered statistically significant.

## 3 Results

### 3.1 PDAC‐PDXs maintain the molecular features of the original tumors and the ability to evoke a desmoplastic reaction

Four PDXs (PDAC‐PDXs Hupa4, Hupa8, Hupa11, and Hupa13), established orthotopically in the pancreas of immunodeficient mice, maintained through serial *in vivo* transplantation, were molecularly and phenotypically characterized.

#### 3.1.1 Major gene mutations in patient specimens are conserved in PDAC–PDXs

**Fig. 1:**
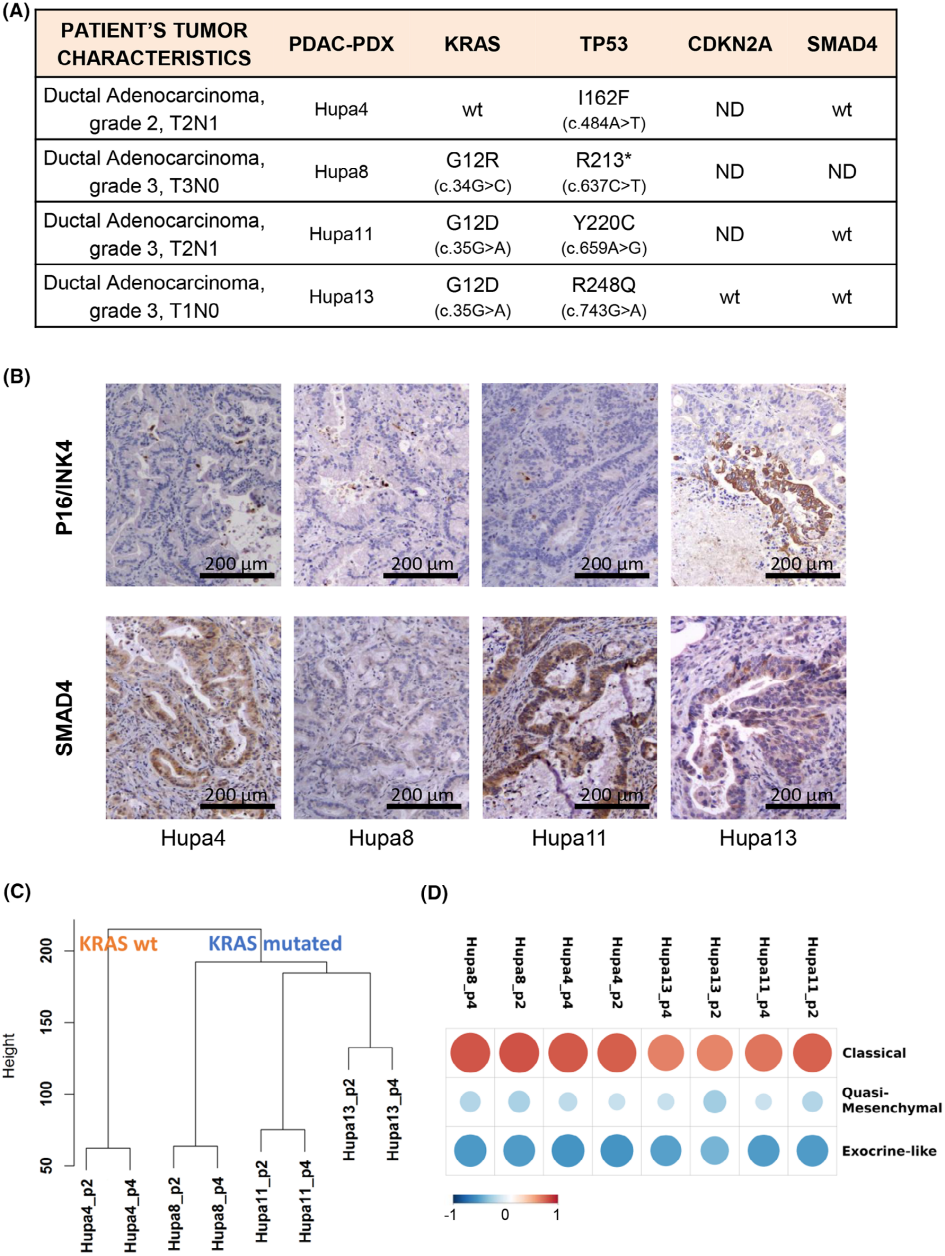
Molecular and histological characterization of orthotopic pancreatic ductal adenocarcinoma patient‐derived xenograft (PDAC‐PDXs: Hupa4, Hupa8, Hupa11, and Hupa13). (A) Tumor features and mutational status of *KRAS*, *TP53*, *CDKN2A*, and *SMAD4* genes. ND: Gene/transcript not detectable [i.e., unable to amplify any of the four *CDKN2A* exons (1a, 1b, 2 and 3) and the *SMAD4* mRNA]. wt, wild‐type. (B) Representative images of PDAC‐PDX tumors immunostained for P16/INK4 and SMAD4. Scale bar: 200 μm. For each PDAC‐PDX, three replicates were evaluated. (C) Unsupervised hierarchical clustering of global gene expression of human tumor compartments. The dendrogram presents the relationship of similarity among the global gene expression profiles of each PDAC‐PDX (average of normalized gene expression data of three replicates) across different *in vivo* passages (P2 and P4). Euclidean distance as distance metric and Ward's as clustering method were used. (D) Subtype classification of PDAC‐PDXs. Centroids were calculated for samples from each class of the Collisson dataset, and then, the Pearson correlation was calculated between centroids and gene expression data from our PDAC‐PDX samples. The correlation matrix shows positive (red dots), or negative (blue dots) correlations with the pancreatic cancer subtypes described by Collisson et al. [13]. Bigger dots correspond to greater positive or negative correlation.

PDAC‐PDXs displayed the mutational pattern typical of PDAC, summarized in Fig. 1A. All four PDAC‐PDXs harbored *TP53* gene mutations, while *KRAS* carried a G12R mutation in Hupa8 and G12D in Hupa11 and Hupa13. *CDKN2A* was not detectable in Hupa4, 8 and 11 which accordingly lacked the encoded p16INK4 protein (Fig. 1B). Only Hupa13 carried the wild‐type (wt) *CDKN2A* gene and, as expected, expressed the p16INK4 protein (Fig. 1B). Hupa4, Hupa11, and Hupa13 carried wt *SMAD4* gene, while *SMAD4* mRNA and protein were not detectable in Hupa8 sections (Fig. 1A,B).

#### 3.1.2 Human gene expression is conserved in PDAC‐PDXs through *in vivo* passages

The mutational status of PDAC‐PDXs was retained in the second and fourth generations of engrafted tumors, along with global gene expression. Genome‐wide expression analysis of tumor cells in PDAC‐PDXs was investigated with human‐specific Agilent microarrays. Unsupervised hierarchical clustering of their human transcriptional profiles indicated that the global gene expression of the PDAC‐PDXs remained stable through *in vivo* passages. Interestingly, the PDAC‐PDXs carrying mutated KRAS clustered more closely, far from the wt one (Hupa4) (Fig. 1C).

Taking the Collisson classification of pancreatic cancers as reference [13], the four PDAC‐PDXs clearly fall within the classical subtypes (positive correlation), robustly excluding the quasi‐mesenchymal and the exocrine subtypes (negative correlation) (Fig. 1D).

#### 3.1.3 PDAC‐PDXs retain primary tumor morphology and an intense stromal reaction

**Fig. 2:**
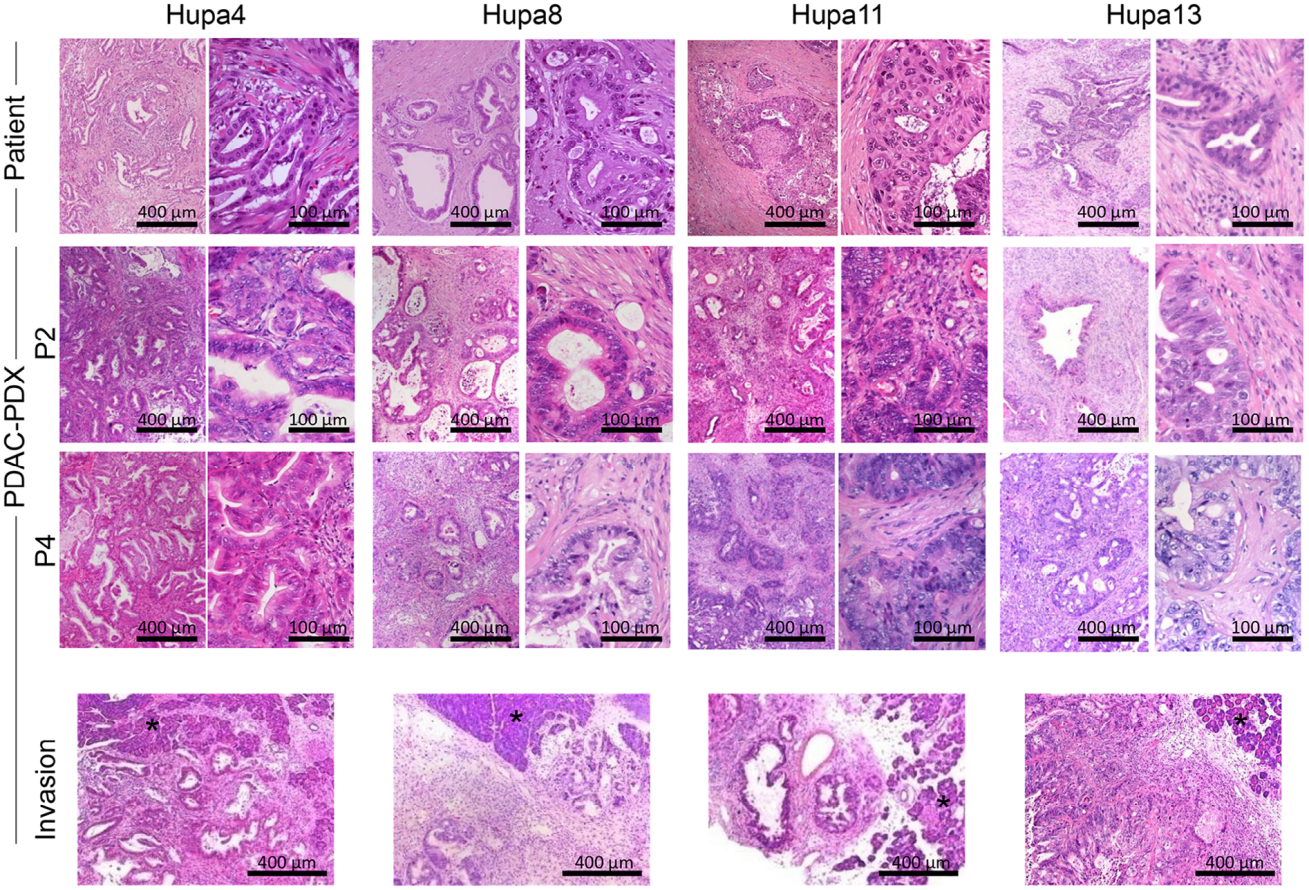
Morphology of pancreatic ductal adenocarcinoma patient‐derived xenograft (PDAC‐PDXs) compared with patients' samples. Representative images of hematoxylin–eosin (H&E) staining of patients' tumor (two different magnifications) and the corresponding PDAC‐PDXs at early (P2) and late passages (P4). In the lower panel, tumor xenografts invading the normal pancreatic tissue (highlighted by the asterisks) of the recipient mice are shown. Scale bar: 400 or 100 μm as indicated. For each PDAC‐PDX three replicates were evaluated.

**Fig. 3:**
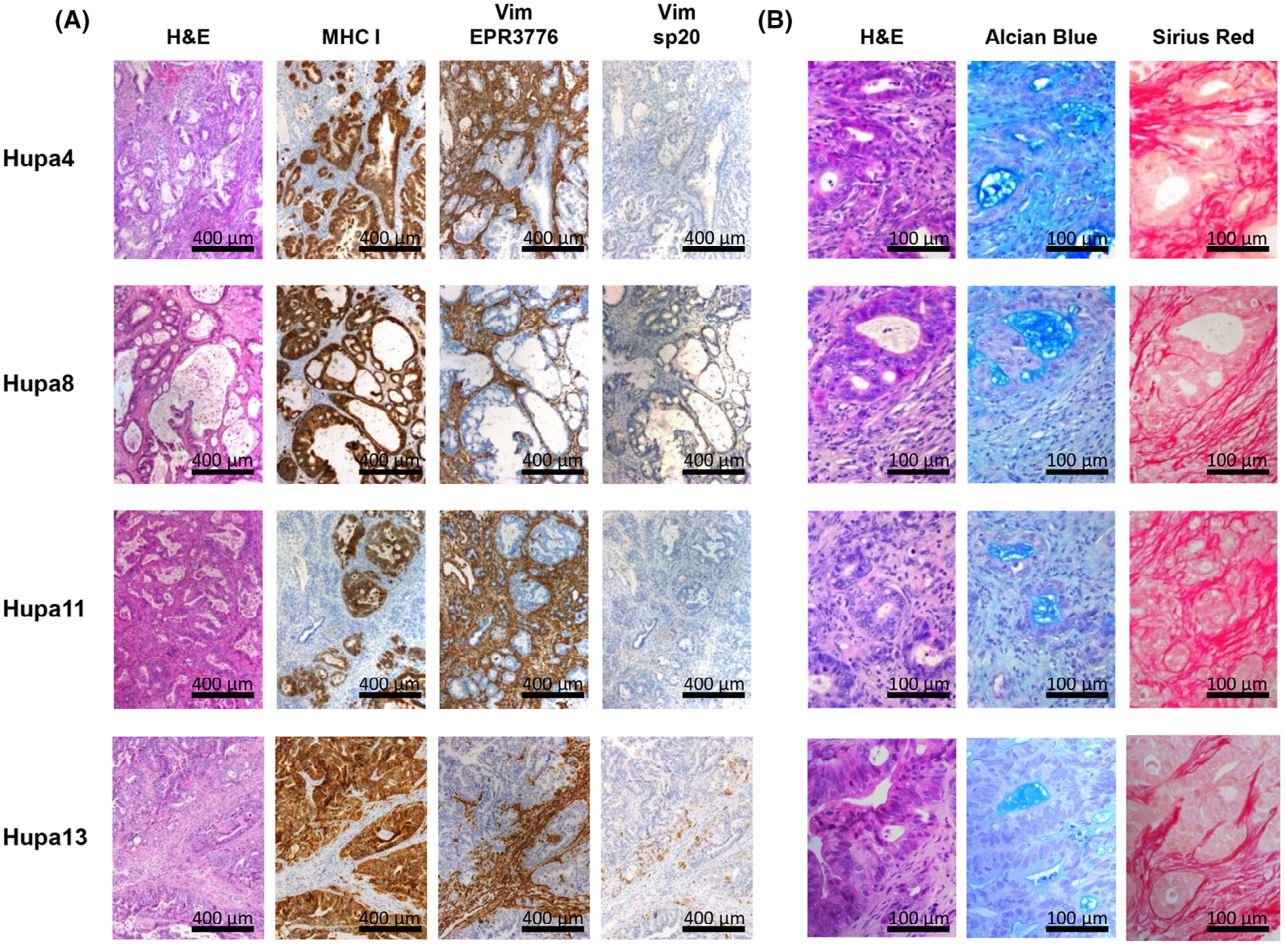
High stroma deposition in pancreatic ductal adenocarcinoma patient‐derived xenograft (PDAC‐PDXs). (A) Representative images of PDAC‐PDX tumor staining for hematoxylin–eosin (H&E), for MHC I (HLA, detecting human tumor parenchyma), for Vimentin EPR3776 (Vim EPR3776, recognizing both the human and murine epitopes), and for Vimentin sp20 (Vim sp20, recognizing the human epitope) to visualize murine stroma. Scale bar: 400 μm. (B) Representative staining for H&E, Alcian Blue (for secretory parenchyma) and Sirius Red (to detect fibrosis). Scale bar: 100 μm. For each PDAC‐PDX, three replicates were evaluated.

The morphology and tissue architecture of the corresponding patient's tumor were maintained in all the orthotopically implanted xenografts and across different passages in the mouse (Fig. 2). Notably, PDAC‐PDXs maintained the invasive behavior typical of PDAC. Engrafted tumors were able to invade and progressively replace the mouse pancreas (Fig. 2, lower panels). As shown in Fig. 3A, the presence of human tumor cells was evidenced by HLA immunohistochemistry (MHC I), while the tumor stroma was almost completely replaced by the host murine cells. Indeed, the tissue sections did not stain for the human‐specific Vimentin sp20 clone antibody but stained positive for Vimentin EPR3776 antibody which recognizes both human and murine Vimentin. Only Hupa13 showed positive staining for human Vimentin in occasional tumor cells, indicative of epithelial mesenchymal transition.

The positive staining for Alcian Blue indicates that the tumors maintained the secretory activity typical of the ductal component of the normal pancreas, while the intense staining for Sirius Red (used for collagen detection) indicates a strong stromal reaction and the fibrosis typical of the patient's PDAC histopathology (Fig. 3B).

### 3.2 PDAC‐PDXs respond heterogeneously to chemotherapy

**Fig. 4:**
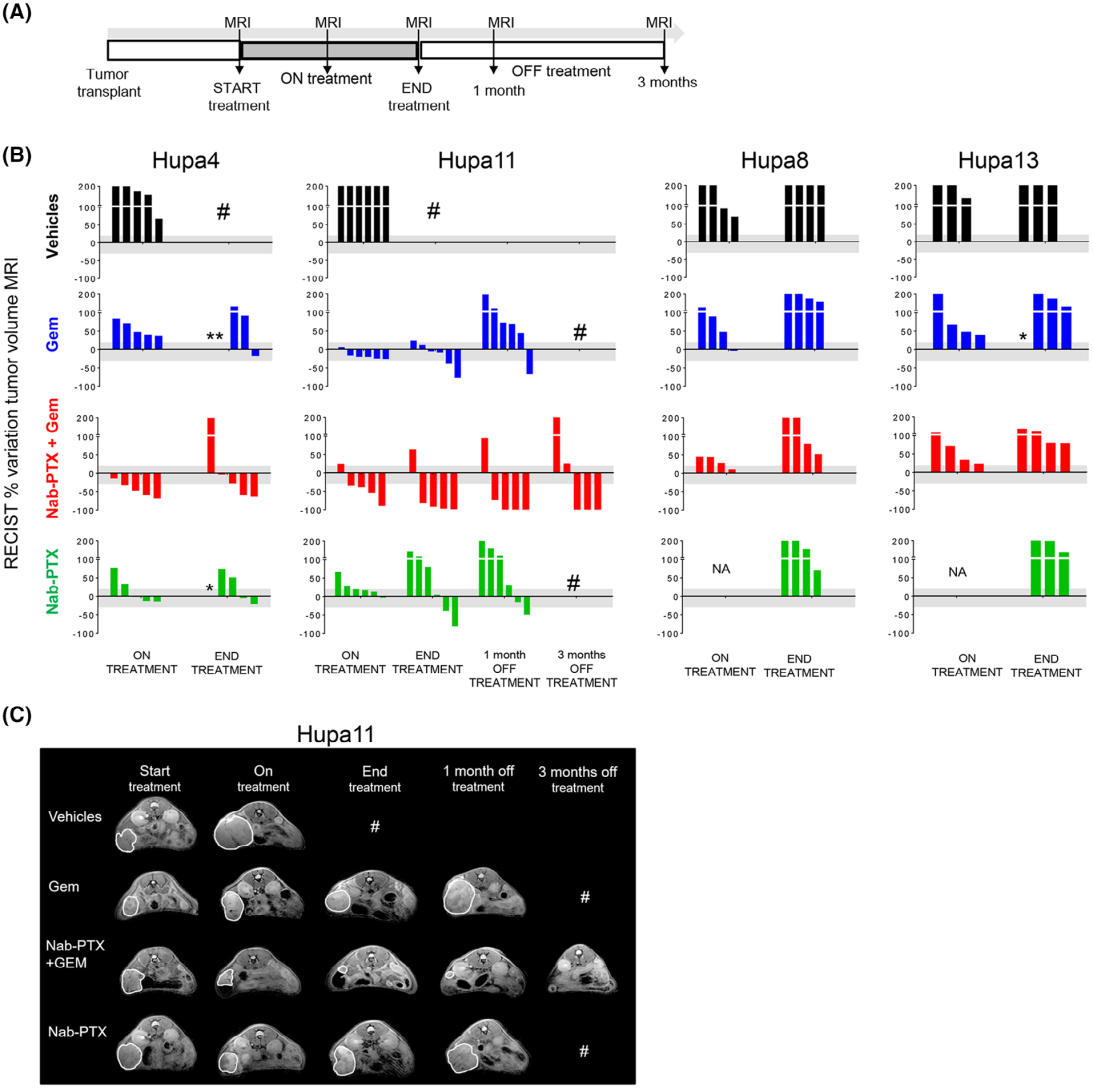
Pancreatic ductal adenocarcinoma patient‐derived xenograft (PDAC‐PDXs) respond differently to the combination of Nab‐PTX and gemcitabine (Gem). (A) Schematic experimental design: tumor fragments of each PDAC‐PDX were implanted into the pancreas of C.B‐17 SCID female mice. When tumors were palpable, magnetic resonance imaging (MRI) was used to evaluate growth. Tumors were randomized at a mean volume of 320 mm^3^ (Standard Deviation: SD, 152) Hupa4, 200 mm^3^ (SD 144) Hupa11, 252 mm^3^ (SD 151) Hupa8, and 233 mm^3^ (SD 93) Hupa13 to receive the different treatments. Gem and/or Nab‐PTX were injected intravenously at respectively 150 and 25 mg·kg^−1^. When administered in combination, Nab‐PTX was delivered immediately before Gem. Drugs were given on Days 1 and 8 of each 21‐day cycle, for a total of four cycles. Tumor growth was monitored over time by MRI and tumor volume was calculated. (B) Drug effect is shown as the percentage change in the tumor volume (calculated as described in Section 2) for each mouse, against time. According to the RECIST guidelines 24, disease is considered progressive if the increase in tumor volume is greater than 20%, stable (gray area in the graphs) if the change is between +20% and −30%, and regressive if the change is lower than −30%. *Single mouse euthanized; ^**#**^all mice in the experimental group euthanized. NA, not available. (C) Hupa11 representative MRI images. The white lines indicate tumor masses. ^**#**^All mice in the experimental group euthanized.

The chemosensitivity of the PDAC‐PDXs was investigated by transplanting Hupa4, Hupa11, Hupa8, and Hupa13 orthotopically into the pancreas of immunodeficient mice and giving them gemcitabine (Gem, 150 mg·kg^−1^) or Nab‐Paclitaxel (Nab‐PTX, 25 mg·kg^−1^) as single treatments or combined. Doses and schedules were calculated in line with recommendations for patients [28]. Tumor response was monitored by MRI throughout the treatment (Fig. 4A).

Gem was weakly effective in controlling tumor growth, stabilizing the disease only in Hupa11 which, however, resumed growth as soon as therapy stopped (Fig. 4B,C). The addition of Nab‐PTX greatly improved the efficacy of Gem against Hupa4 and Hupa11, but not against Hupa8 and Hupa13, reflecting the heterogeneous response of PDAC to chemotherapy. There was in fact robust tumor regression in four out of five Hupa4 and Hupa11 bearing mice. In particular, three out of five Hupa11 bearing mice were cured 3 months after withdrawal of therapy, as confirmed by the absence of disease at autopsy (Fig. 4B,C).

Nab‐PTX as single agent had limited activity. Tumor stabilization or regression at the end of treatment was achieved only in two out of five Hupa4 and three out of six Hupa11.

As there were no significant differences in tumor growth in Hupa8 and Hupa13 bearing mice treated with vehicle, Gem, or Nab‐PTX as single agents or combined, the four groups of mice were euthanized immediately after the end of therapy.

The pattern of response of Hupa11 (responsive) and Hupa8 (not responsive) was similar using the combination of Gem with another formulation of paclitaxel (cremophor EL‐PTX) (Fig. S1).

#### 3.2.1 The responsiveness to gemcitabine is mediated by the stroma compartment

**Fig. 5:**
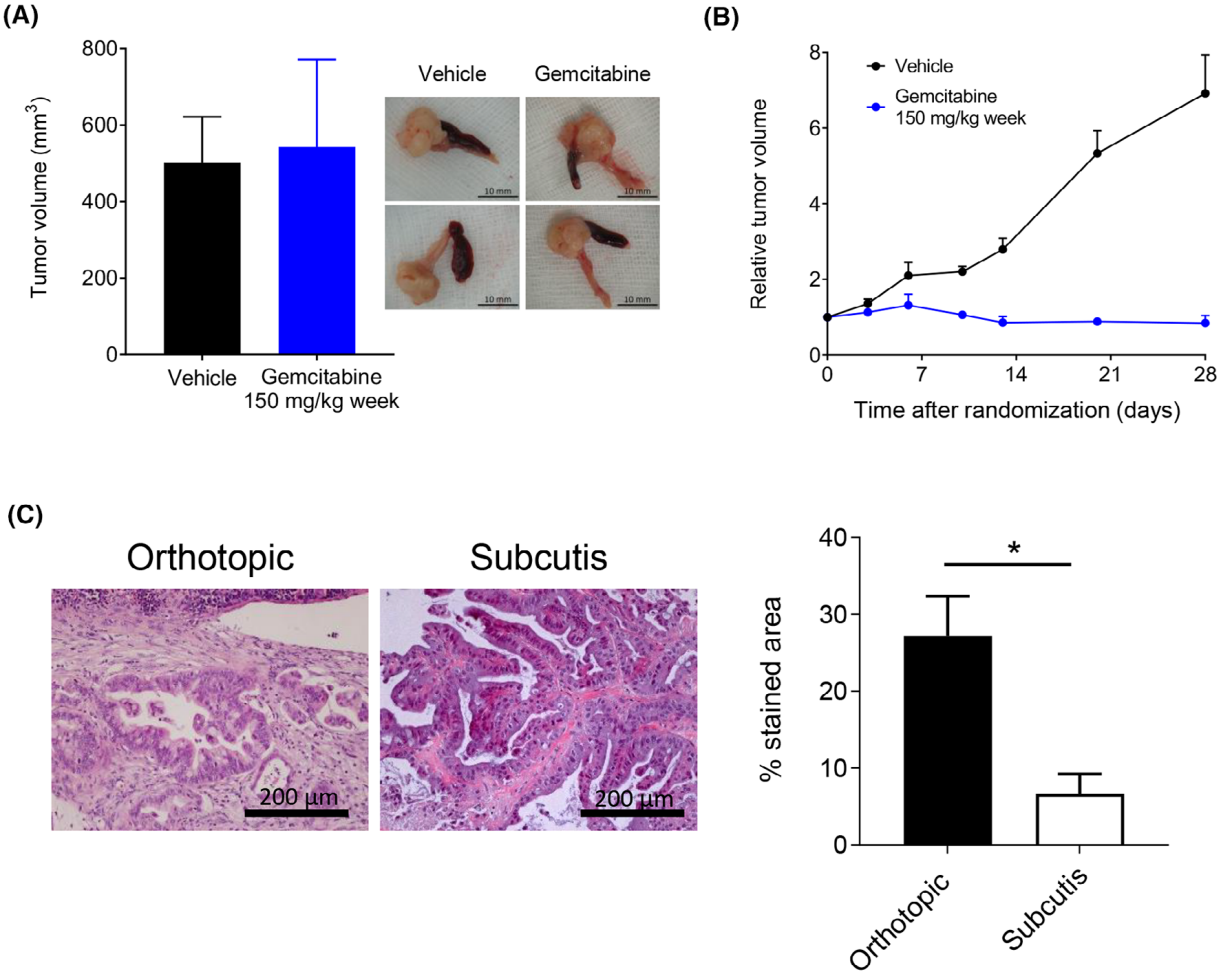
Contribution of the microenvironment to pancreatic ductal adenocarcinoma patient‐derived xenograft (PDAC‐PDX) responsiveness to chemotherapy. (A, B) Hupa4 tumor fragments were implanted into the pancreas (A) and subcutis (B) in C.B‐17 SCID female mice. Tumor growth was monitored over time by palpation for the intrapancreas tumor and by measurements with Vernier calipers (see Section 2) for the subcutaneous tumor. When subcutis tumors reached 275 mm^3^ (Standard Deviation: SD, 107) mice were randomized (four to five per group) into treatment, and likewise for the intrapancreas tumor when they were all palpable. Gemcitabine was injected intravenously at the dose of 150 mg·kg^−1^ on Days 1 and 8 of each 21‐day cycle for two cycles. For both settings, intrapancreas tumor weights (mean ± standard error mean, SEM), with representative images at autopsy (A), and relative tumor volume curves for subcutaneous growth (B) are shown (for each point mean ± SEM is shown). Scale bar: 10 mm. (C) Representative images of hematoxylin and eosin (H&E) staining of intrapancreas and subcutis Hupa4 tumors. Quantitative analysis of Sirius Red staining is shown and data are expressed as the percentage of stained area (mean ± SD, *n* = 3 for each setting). *P < 0.05, unpaired *t*‐test. Scale bar: 200 μm.

To investigate whether the response to chemotherapy was due to the intrinsic responsiveness of the tumor cells or was influenced by the microenvironment, gemcitabine therapeutic effect was examined in tumors growing orthotopically or ectopically (subcutis). Orthotopically growing Hupa4 tumors did not respond to Gem 150 mg·kg^−1^ (Fig. 5A), while Hupa4 transplanted subcutis and treated with the same regimen achieved stabilization (Fig. 5B). Results were similar with Gem 300 mg·kg^−1^ (Fig. S2A,B), indicating that the lack of response cannot be ascribed to intrinsic characteristics of the cancer cells, and rather suggesting a role of the host tumor microenvironment in the response to chemotherapy. Hupa4 transplanted in the mouse pancreas evoked a stronger stromal reaction than Hupa4 transplanted subcutis, supporting this hypothesis (Fig. 5C). These results were confirmed with Hupa11, which resulted highly sensitive to Gem when transplanted subcutis (Fig. S3A), but only partially responsive when transplanted orthotopically in the pancreas (Fig. 4B). Vimentin and Sirius Red staining quantification highlighted a stronger stromal reaction in Hupa11 tumors growing in the mouse pancreas than in tumors growing subcutis (Fig. S3B) as observed in Hupa4 (Fig. 5C).

### 3.3 Responsiveness to chemotherapy is associated with a specific stroma transcriptional signature

#### 3.3.1 Transcriptomic analysis identifies a 294‐gene signature distinguishing PDAC‐PDX responses‐to‐treatment subtypes

**Fig. 6:**
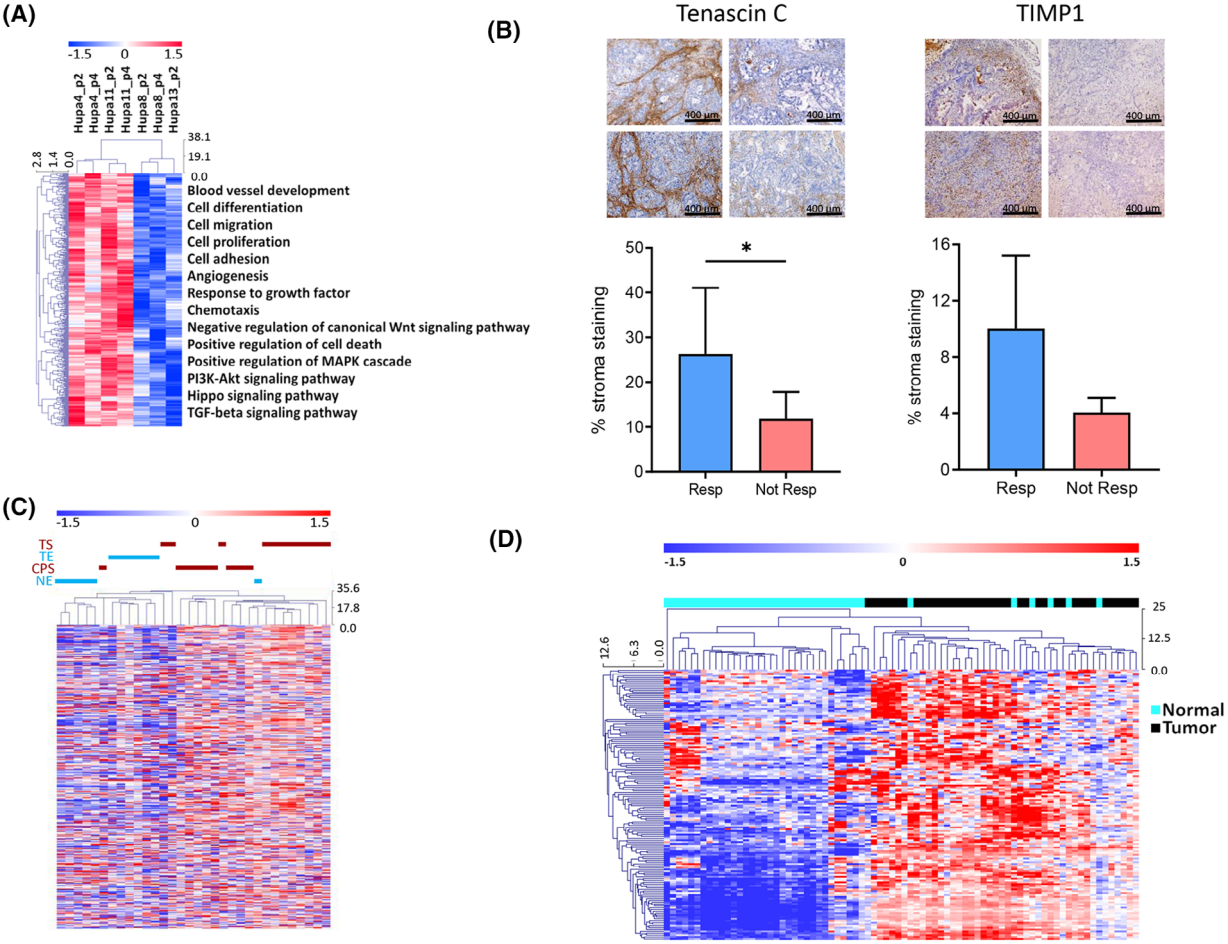
Stroma signature. (A) Heatmap presenting the 294 upregulated genes in murine cancer microenvironment of responsive (Resp) and not responsive (Not Resp) pancreatic ductal adenocarcinoma patient‐derived xenografts (PDAC‐PDXs). Gene Ontology processes and pathways with statistically significant enrichment are reported. (B) Representative images and quantification (mean ± standard error mean, *n* = 8 for each setting) of immunohistochemical staining for Tenascin‐C and TIMP1 in responsive (Hupa4 and Hupa11) vs not responsive (Hupa8 and Hupa13) PDAC‐PDXs. *P < 0.05, unpaired *t*‐test. Scale bar: 400 μm. (C) Heatmap presenting the specificity of the stroma signature in a microdissected Pilarsky dataset (E‐MEXP‐950/E‐MEXP‐1121). CPS, pancreatitis fibrotic stroma; NE, normal epithelium; TE, tumor epithelium; TS, tumor stroma. (D) Heatmap of the stroma signature in a ‘whole tissue’ clinical dataset (GSE15471, Badea), confirming the ability to distinguish healthy from tumor tissues. For all four heatmaps, *Z*‐scores (median‐centered log_2_ intensity divided by standard deviation) are reported by a red‐to‐blue gradient to indicate up‐ or downregulation for each gene. Unsupervised hierarchical clustering of samples was based on the *Z*‐scores.

To identify differences in stroma features, mouse‐specific microarrays were used to investigate PDAC‐PDX gene expression, having proved that the tumor microenvironment originated from the murine host (see above Fig. 3A). To find characteristics predicting benefit from the combination treatment with Gem + Nab‐PTX, we searched for the genes differently expressed in responsive *vs* not responsive PDAC‐PDX stroma. Class comparisons analysis revealed significantly higher expression of 294 genes in responsive than in not responsive subset, most belonging to the extracellular region and the membrane compartment (*n* = 206) (Table S2). There were genes encoding for different collagen chains, metallopeptidases, fibrillins, laminin subunits, thrombospondin 1 and 2, cadherin 11 and 13, fibroblast activation protein alpha, and fibroblast growth factor receptor 1 as well as genes related to the immune response like granzymes and mcpt genes. Functional classification analysis of the 294 upregulated genes revealed significant enrichment in biological processes, such as cell differentiation, migration, proliferation, adhesion and chemotaxis, angiogenesis, cell death, and immune response, as well as Wnt, MAPK, PI3‐Akt, Hippo, and TGF‐beta pathways (Fig. 6A). The differential expression of selected transcripts, such as Tenascin‐C and TIMP1 in responsive and not responsive PDAC‐PDX, was confirmed by immunohistochemistry in responsive and not responsive PDAC‐PDXs (Fig. 6B).

#### 3.3.2 The PDXs stroma signature genes are expressed by the stroma of PDAC clinical samples

The 294 genes overexpressed in the microenvironment of responsive PDAC‐PDXs were investigated in clinical samples by interrogating publicly available datasets, and selecting the human orthologs of the mouse genes. As shown in Fig. 6C, the signature, tested by unsupervised hierarchical clustering on the E‐MEXP‐950/E‐MEXP‐1121 dataset [22], robustly discerned the stroma (S) from the epithelial (E) constituents, regardless of their derivation [i.e., whether S and E were microdissected from PDAC (TS and TE), normal ducts of pancreas (NE), or pancreatitis fibrotic tissue (CPS)]. When bulk tissue gene expression was interrogated using the GEO GSE15471 dataset [18], the 294‐gene signature robustly discerned tumors (highly expressing the signature) from matched ‘normal’ tissues of resected pancreas (Fig. 6D), which *per se* is devoid of stroma cellularity.

Altogether, these results imply that the 294‐gene signature is expressed by the tumor microenvironment of the PDAC, thus clinically validating the PDX findings.

#### 3.3.3 The stroma signature distinguishes subsets of PDAC in clinical samples cohorts

**Fig. 7:**
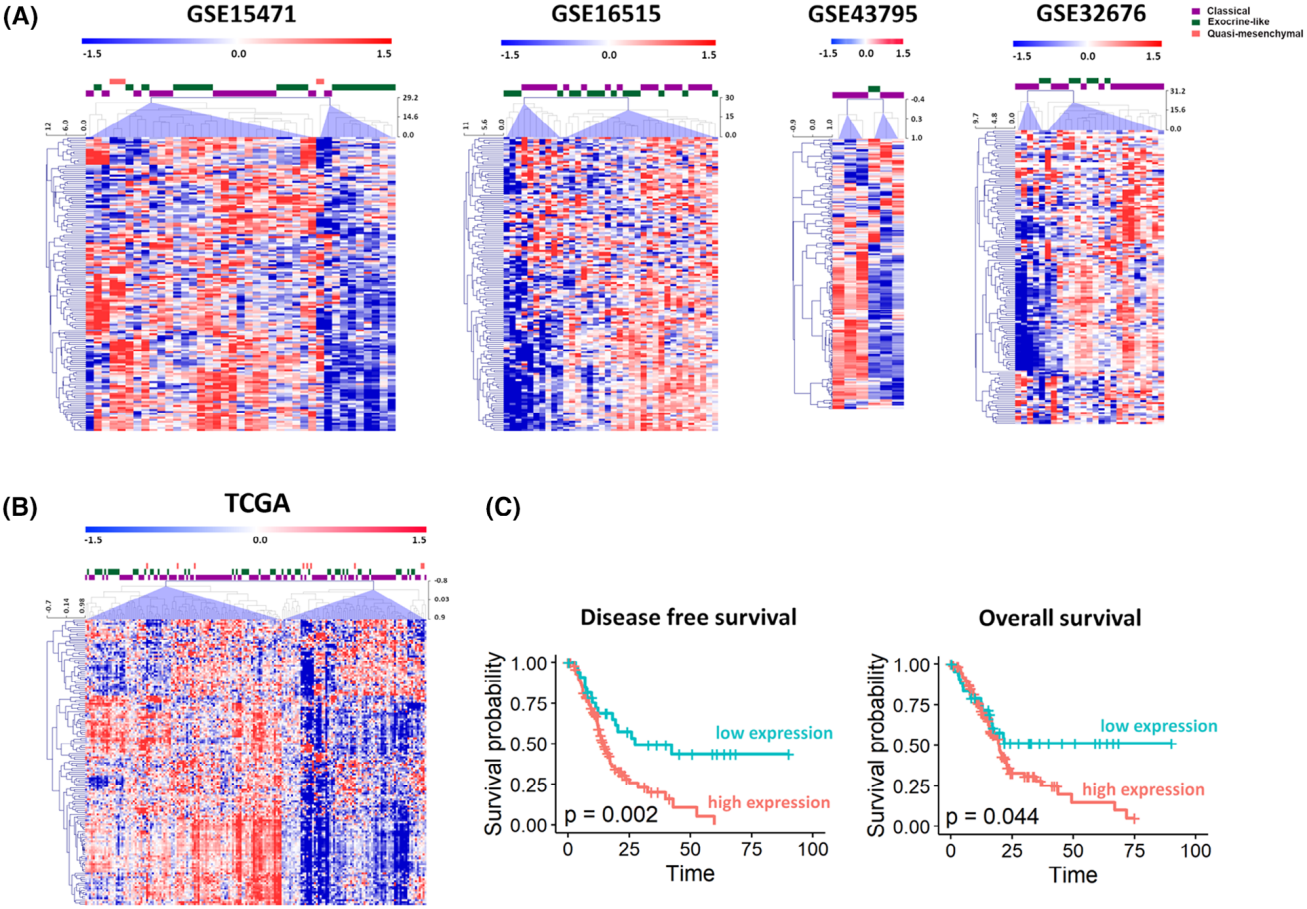
Stroma signature in clinical tumor samples. (A, B) Stroma signature in pancreatic ductal adenocarcinoma (PDAC) samples from GSE15471, GSE16515, GSE43795, and GSE32676 clinical datasets (A) and from TCGA (B). *Z*‐scores (median‐centered log_2_ intensity values divided by standard deviation) are reported by a red‐to‐blue gradient to indicate levels of up‐ or downregulation for each gene. Unsupervised hierarchical clustering of samples was based on the *Z*‐scores. In each dataset, the signature separates tumor samples into two main clusters (lilac triangles), according to their high or low expression. (C) High expression of the stroma signature is associated with reduced disease‐free survival and overall survival in the TCGA dataset. Kaplan–Meier curves are presented. Pancreatic cancer patients were stratified into two groups depending on high (75th percentile) or low (25th percentile) mean expression of the stroma signature. Statistical significance was calculated by the log‐rank test between the groups. The disease‐free Kaplan–Meier curve: low expression (*n* = 35 samples) and high expression (*n* = 103 samples). The overall survival Kaplan–Meier curve: low expression (*n* = 45 samples) and high expression (*n* = 133 samples).

Next, we interrogated five publicly available datasets (GSE15471, GSE16515, GSE43795, GSE32676, and TCGA) to validate the robustness of the 294‐gene signature in distinguishing clinical samples of PDAC. As shown in Fig. 7A,B, the signature is able to cluster and distinguish a patient's PDAC subset, not associated with tumor subtypes following Collisson's classification. Results (Fig. 7C) from The Cancer Genome Atlas (TGCA), a clinical dataset that combines gene expression features and major clinical outcome endpoints, show that in patients the high expression of the 294‐gene signature have worse prognosis, with both worse disease‐free and overall survival.

## 4 Discussion

We molecularly and pharmacologically characterized four PDAC‐PDXs and identified a stroma molecular signature that distinguishes responsive from not responsive PDAC‐PDX to the combination of gemcitabine with Nab‐Paclitaxel.

Four PDAC‐PDXs, established orthotopically in the pancreas of immunodeficient mice, maintained the histological and molecular characteristics of the original tumors through different *in vivo* passages, indicating that they were reliable and reproducible models of human PDAC. PDAC‐PDXs presented distinctive PDAC gene mutations affecting different signaling pathways and cell functions [29].

The most important feature of this study is that the four PDAC‐PDXs were able to re‐create a tumor microenvironment with the original properties of the PDAC tumors in patients. This further supports our recent studies showing that Hupa4, Hupa8, and Hupa11 PDAC‐PDX release high levels of the same stroma‐related molecules as patients. These biomarkers were validated in two separate cohorts of patients and found to be associated with the presence of PDAC, indicating that the stroma present in PDXs is informative of PDAC stroma in patients [15]. Some of these molecules, such as TIMP1, THBS2, ICAM‐1, and IGFBP2, have been analyzed as potential PDAC diagnostic biomarkers in other studies, supporting once again their relevance in humans [30].

Drug resistance is one of the main challenges in treating PDAC, with combined chemotherapy still being the principal treatment option. Therefore, identification of a molecular signature, predicting the response to chemotherapy, would be important to select patients who could potentially benefit from treatments [31]. Our four PDAC‐PDXs showed different responsiveness to chemotherapeutic drugs, particularly to the combination of gemcitabine with Nab‐Paclitaxel (Gem + Nab‐PTX), enabling us to investigate the determinants of drug response.

Significant efforts have been made to define PDAC subtypes with distinct molecular characteristics. Our results, despite the limited number of cases, suggest that the mechanism of resistance is not related to any intrinsic cancer cell features. Global gene expression showed that all the four different PDAC‐PDXs belong to the ‘classical subtype’ of PDAC described by Collisson et al. [13]. Differences in human transcriptome (Fig. 1C) did not correlate with drug response, nor was drug response related to the presence of KRAS mutations.

Experiments with Hupa4 PDX revealed that cancer cells were responsive to gemcitabine when grown subcutaneously, but not when grown intrapancreas, highlighting the tumor microenvironment as a potential influencer of drug response. Previous studies have shown the crucial role of stroma in chemotherapy resistance in PDAC [32]. It has been postulated that the intensive fibrosis and the large amount of stroma might constitute a physical barrier, hindering drug penetration into the tumors [33]. As disproof of this, no significant differences in stroma abundance between the different PDAC‐PDXs were detected as shown by Vimentin and Sirius Red staining quantification across multiple samples (Fig. S4). Moreover, we demonstrated by Mass Spectrometry Imaging (MSI) that the lack of tumor response was not due to poor penetration or to different distribution of PTX in not responsive compared with responsive tumors. The pharmacokinetic profiles of PTX measured by HPLC in tumor and plasma of PDAC‐PDXs with different responsiveness to the drug were similar, once again indicating no significant difference in drug penetration in the tumors and the absence of systemic pharmacokinetic effects (Fig. S5).

Although we found comparable pharmacokinetic profiles and intratumor drug distribution of PTX in responsive and not responsive PDAC‐PDX, the difference in combination regimens compared with gemcitabine monotherapy was more pronounced. A possible effect of Nab‐PTX on tumor stroma leading to an increase in intratumor gemcitabine concentration in tumors expressing high levels of collagen cannot be excluded [34]. We speculate that in Hupa4 and Hupa11, the higher expression of collagens might explain the greater effectiveness of Nab‐PTX combined with gemcitabine. By collapsing collagen fibers, Nab‐PTX could indeed raise the gemcitabine intratumor concentration and activity. Tumor stiffness reflects differences in extracellular matrix composition, and elastography has been suggested to assess the stromal disrupting effects of Nab‐PTX in PDAC patients [35]. Further pharmacokinetic studies are warranted to confirm this.

The ‘294‐gene stroma signature’ associated with the response to Gem + Nab‐PTX was robustly validated. Hupa4 and Hupa11, expressing high levels of the stroma genes identified, are responsive to Gem + Nab‐PTX, while low‐expressing Hupa8 and Hupa13 did not gain any benefit from the treatment.

In accordance with the hypothesis that drug response may be influenced by the specific pancreas microenvironment characteristics, we found that a different expression of 294 stroma genes was potentially associated with drug response. Several of the genes overexpressed in responsive compared with not responsive PDAC‐PDXs belong to pathways previously described as mediating the resistance to gemcitabine in PDAC [36]. These pathways include genes that affect drug metabolism, activate antiapoptotic pathways, create a hypoxic and acidic microenvironment, influence epithelial‐to‐mesenchymal transition, cancer‐associated fibroblast and immune cell activation, protease expression, and extracellular matrix remodeling.

By selecting the most differentially expressed genes in terms of *P*‐value and logFoldChange from the 294 genes of the signature, we found a sub‐signature of 24 genes, including LRRC15, Tenascin‐C, Integrin‐β1 ligand, Itgb1bp2, and Collagens, Col6a1 and Col6a2, and Granzyme e, c, d, that have been described to have a role in pancreatic cancer and could help to gain insights into the mechanisms of response/resistance to therapy (Fig. S6A) [37, 38, 39, 40].

Interestingly, the 294‐gene signature comprised genes that are among the ‘activated’ stroma genes defined by Moffitt [41]. Hierarchical clustering of the samples using the ‘activated’ and ‘not activated’ stroma genes defined by Moffitt separated the PDAC‐PDXs into two distinct subsets, with either high (Hupa4 and Hupa11) or poor (Hupa8 and Hupa13) expression of the activated stroma genes (Fig. S6B). These data, on the one hand, validate the high expression of the ‘activated stroma genes’ as a signature of chemotherapy response and, on the other hand, support once again our PDXs as valid preclinical models to study drug response in PDAC.

The analysis of human datasets showed that the 294‐gene signature can distinguish stroma‐rich tumors from matched normal pancreas devoid of stroma (GSE15471), confirming the validity of PDX models and the robustness of the signature. In addition, the 294 stroma gene signature distinguishes patients with worse overall and disease‐free survival. This is particularly important since it could potentially be used to select a subset of patients with particularly aggressive tumors which, however, might well benefit from treatment with Gem + Nab‐PTX. Interestingly, a class comparison analysis of the TCGA dataset, focusing on KRAS‐mutated versus nonmutated samples revealed that 44% of the genes in the stroma signature exhibited statistically significant differential expression (adjusted *P*‐value < 0.05) based on the mutation status. This suggests a potential relationship between this driver mutation and the stroma gene signature, warranting further investigation.

Unfortunately, there are no accessible datasets of pancreatic adenocarcinomas that contain detailed therapy information, particularly regarding the response rate to Gem + Nab‐PTX treatments. While some studies have explored survival outcomes and disease progression, datasets specifically stratifying patients by treatment, particularly distinguishing between chemo‐treated and chemo‐naïve patients, are lacking in public repositories, preventing our ability to validate the predictive value of the 294‐gene signature in patients.

## 5 Conclusions

We validated our PDAC‐PDXs as reliable sources of information regarding drug responsiveness. Specifically, we defined a stroma molecular signature in PDAC‐PDX associated with chemotherapy response that faithfully represents activated stroma in patients. Further clinical validation is needed to confirm its utility in selecting patients potentially responsive to Gem + Nab‐PTX, avoiding patients' exposure to the toxic effects of treatments that do not offer much survival benefit.

## Conflict of interest

The authors declare no conflict of interest.

## Author contributions

RG, CG, and DB contributed to the conception and design. AA, LF, PO, LuM, AR, LaM, CF, EM, and CM contributed to the development of methodology. AA, LF, PO, LuM, AR, LaM, CF, EM, CM, ES, GC, RG, CG, and DB contributed to the data analysis and interpretation. PO, GC, RG, CG, and DB contributed to the writing/review of the manuscript. RG, CG, and DB contributed to the study supervision. All authors read and approved the final manuscript.

## Supporting information


**Fig. S1.** PDAC‐PDX response to cremophor EL‐paclitaxel (crem‐PTX) and combined with Gemcitabine (Gem).
**Fig. S2.** Gemcitabine double dose did not improve response to therapy.
**Fig. S3.** Microenvironment contribution to HuPa11 responsiveness to chemotherapy.
**Fig. S4.** Quantification of stroma abundance in pancreatic ductal adenocarcinoma patient‐derived xenografts (PDAC‐PDXs).
**Fig. S5.** PDAC‐PDX responsiveness is not associated with a different pharmacokinetic profile or intratumor distribution of paclitaxel (PTX).
**Fig. S6.** Clustering of PDAC‐PDXs based on expression of 24‐stroma gene sub‐signature and Moffitt‐activated stroma genes.

**Table S1.** Primers used for PCR amplification and sequencing and annealing temperature for each primer pair are indicated.

**Table S2.** List of upregulated genes in samples from responsive (Hupa4 and Hupa11) or not responsive tumors (Hupa8 and Hupa13). Enrichment in biological processes and molecular functions from Gene Ontology, together with pathways, are also reported.

## Data Availability

Raw and processed data are deposited on the GEO database (GSE234259).
