# Supplementary material for: Stroma gene signature predicts responsiveness to chemotherapy in pancreatic ductal adenocarcinoma patient‐derived xenograft models
Source: Mol Oncol. 2025 Feb 4;19(4):1075–91. doi: 10.1002/1878-0261.13816 (PMC11977644; doi:10.1002/1878-0261.13816)
Supplement: Supplementary file 1 — Fig. S1. PDAC‐PDX response to cremophor EL‐paclitaxel (crem‐PTX) and combined with Gemcitabine (Gem). Fig. S2. Gemcitabine double dose did not improve response to therapy. Fig. S3. Microenvironment contribution to HuPa11 responsiveness to chemotherapy. Fig. S4. Quantification of stroma abundance in pancreatic ductal adenocarcinoma patient‐derived xenografts (PDAC‐PDXs). Fig. S5. PDAC‐PDX responsiveness is not associated with a different pharmacokinetic profile or intratumor distribution of paclitaxel (PTX). Fig. S6. Clustering of PDAC‐PDXs based on expression of 24‐stroma gene sub‐signature and Moffitt‐activated stroma genes. [file MOL2-19-1075-s003.zip › FiguresS1-S6_Legends.docx]

**Supplementary Figure S1. Pancreatic ductal adenocarcinoma patient-derived xenograft (PDAC-PDX) response to** **cremophor EL-paclitaxel (crem-PTX) and combined with Gemcitabine (Gem)**

Tumor fragments were implanted in the pancreas of C.B-17 SCID female mice. Tumor-bearing mice underwent noninvasive magnetic resonance imaging (MRI) randomization (tumor volume 199 mm^3^ (Standard Deviation - SD - 92) Hupa11 and 272 mm^3^ (SD 153) Hupa8). These treatment groups were divided as in the experiment reported in Figure 4 (which also shows vehicle and other treatment groups). Gem and/or crem-PTX were injected intravenously at the doses of 150 mg/kg and 25 mg/kg respectively. When injected in combination crem-PTX was delivered immediately before Gem. Drugs were given on days 1 and 8 of each 21-day cycle for a total of four cycles. Tumor growth was monitored over time by MRI (Figure 4) and tumor volume was calculated as described in Materials and Methods.

Results are shown as the percentage change in tumor volume for each mouse, against time. According to the RECIST guidelines [42], disease is considered progressive if the increase of tumor volume is greater than 20%, stable (grey area in the graphs) if the change is between + 20% and - 30%, tumor shrinkage goes below -30%.

NA: not available; (*) one mouse euthanised; (**#**) all mice in the group euthanised.

**Supplementary Figure S2. Gemcitabine double dose did not improve response to therapy**

A-B. Hupa4 tumor fragments were implanted into the pancreas (A) and subcutis (B) in C.B-17 SCID female mice. Tumor growth was monitored over time by palpation for intrapancreas tumor and by measurements with Vernier calipers (see Materials and Methods) for subcutaneous tumor. Mice were randomized (4-5 per group) when subcutis tumors reached 295mm^3^ (Standard Deviation - SD - 115), and the intrapancreas tumors were all palpable, to receive gemcitabine 300 mg/kg or vehicle on days 1 and 8 of each 21-day cycle for two cycles. For both settings, intrapancreas tumor weights (mean ± standard error mean - SEM), with representative images at autopsy (A), and relative tumor volume curves for subcutaneous growth (B) are shown (for each point mean ± SEM is shown). Scale bar: 10 mm.

**Supplementary Figure S3. Microenvironment contribution to HuPa11 responsiveness to chemotherapy**

A. Hupa11 tumor fragments were transplanted subcutis in C.B-17 SCID female mice. Tumor growth was monitored over time by measurements with Vernier caliper. When tumors reached 368mm^3^ (Standard Deviation – SD - 167) mice were randomized (5 per group) into treatment. Gemcitabine was injected intravenously at the dose of 150 mg/kg on days 1 and 8 of each 21-day cycle for four cycles. Relative tumor volume curves are shown. Data are represented as mean ± standard error mean.

B. Quantitative analysis of Vimentin and Sirius Red staining is shown. Data are expressed as the percentage of stained area (mean ± SD, n=3 for each setting). *p<0.05, unpaired t-test.

**Supplementary Figure S4. Quantification of stroma abundance in pancreatic ductal adenocarcinoma patient-derived xenografts (PDAC-PDXs)**

Quantitative analysis of Sirius Red staining (A) and Vimentin (B) is shown and data are expressed as the percentage of stained area (mean ± SD, n=5 for each setting). ns, unpaired t-test.

**Supplementary Figure S5. Pancreatic ductal adenocarcinoma patient-derived xenograft (PDAC-PDX) responsiveness is not associated to a different pharmacokinetic profile or intratumor distribution of paclitaxel (PTX)**

A. Mice bearing orthotopic tumors (n=8-9/group) were injected with 60 mg/kg of Nab-PTX intravenously and euthanised 4h after treatment. The PTX concentration measured by HPLC in tumor masses and plasma, and the ratio between tumor and plasma (mean ± standard error mean - SEM) is shown. Not responsive (Hupa8 and Hupa13) and responsive (Hupa4 and Hupa11) PDAC-PDXs were compared.

B. MALDI imaging mass spectrometry of representative tumors.

C. PTX distribution expressed as the percentage of positive pixels above the threshold (mean ± SEM).

**Supplementary Figure S6*.* Clustering of pancreatic ductal adenocarcinoma patient-derived xenografts (PDAC-PDXs) based on expression of 24-stroma gene sub-signature and Moffit-activated stroma genes**

A. Heatmap of the 24 top differentially expressed genes (24-gene sub-signature) in responsive and not responsive PDAC-PDXs. Col26a1 and Tnc gene expression was evaluated using two different probes.

B. PDAC-PDX expression profile of the “Normal” or “Activated” stroma genes described by Moffit et al. [41].
