# Supplementary figures and images for: Stroma gene signature predicts responsiveness to chemotherapy in pancreatic ductal adenocarcinoma patient‐derived xenograft models
Source: Mol Oncol. 2025 Feb 4;19(4):1075–91. doi: 10.1002/1878-0261.13816 (PMC11977644; doi:10.1002/1878-0261.13816)

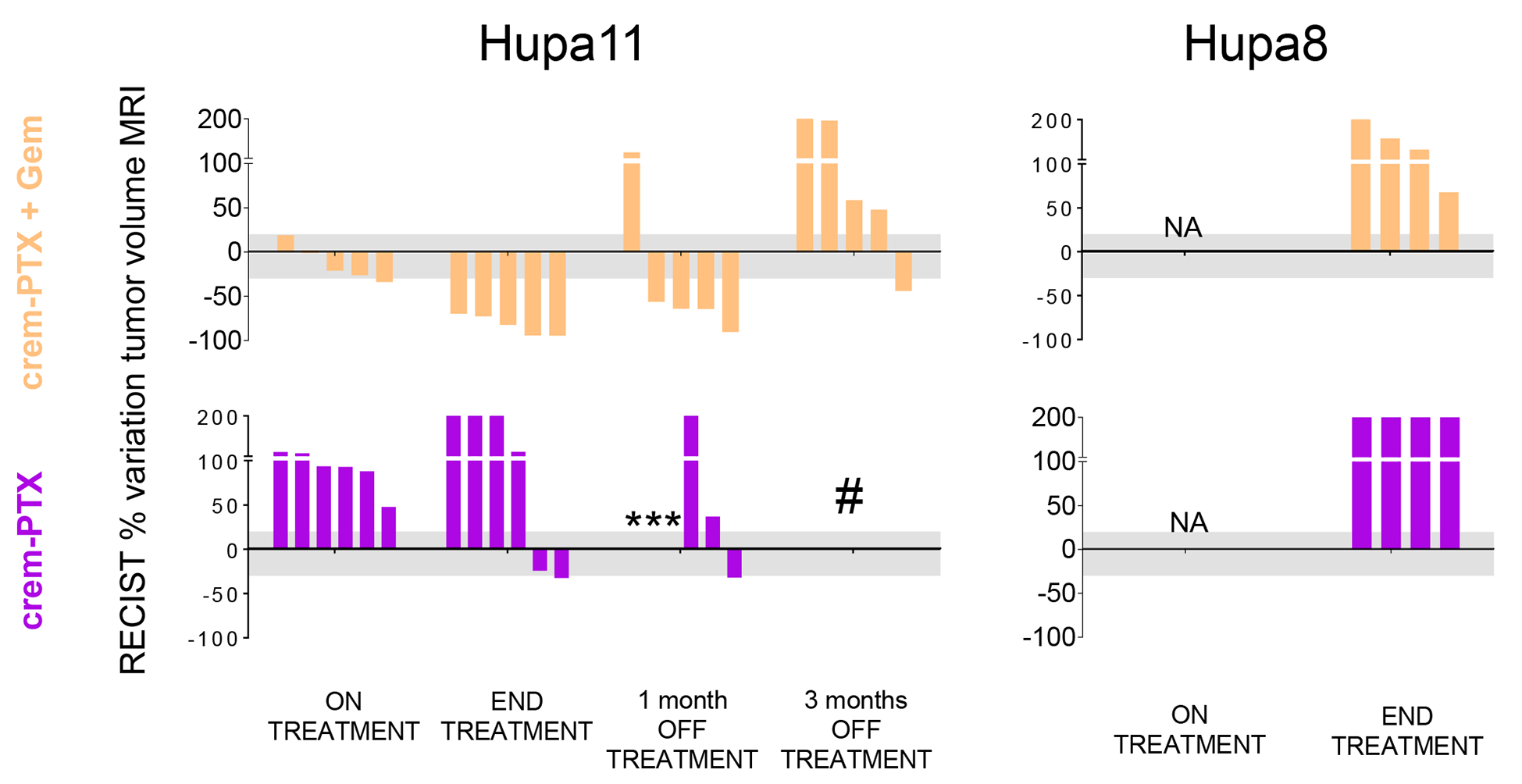

Supplement: Supplementary file 1 — Fig. S1. PDAC‐PDX response to cremophor EL‐paclitaxel (crem‐PTX) and combined with Gemcitabine (Gem). Fig. S2. Gemcitabine double dose did not improve response to therapy. Fig. S3. Microenvironment contribution to HuPa11 responsiveness to chemotherapy. Fig. S4. Quantification of stroma abundance in pancreatic ductal adenocarcinoma patient‐derived xenografts (PDAC‐PDXs). Fig. S5. PDAC‐PDX responsiveness is not associated with a different pharmacokinetic profile or intratumor distribution of paclitaxel (PTX). Fig. S6. Clustering of PDAC‐PDXs based on expression of 24‐stroma gene sub‐signature and Moffitt‐activated stroma genes. [file MOL2-19-1075-s003.zip › MOL213816-sup-0001-FigS1.tif]

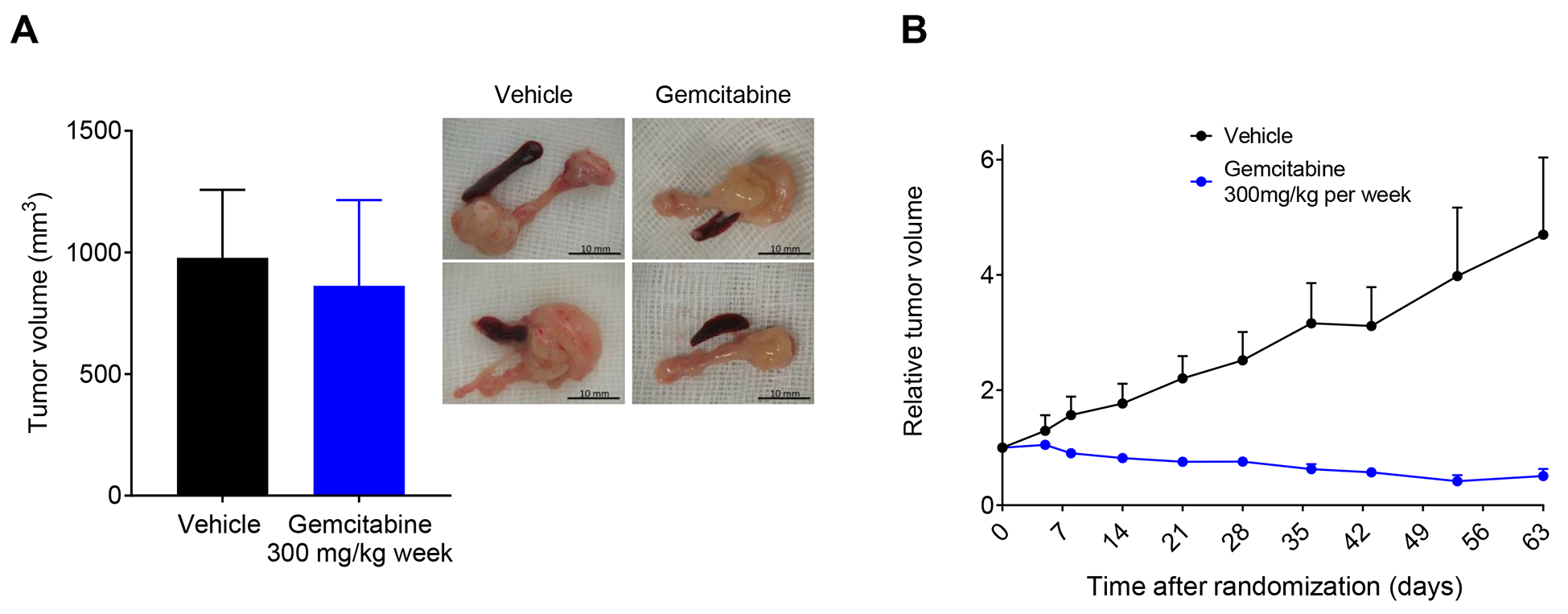

Supplement: Supplementary file 1 — Fig. S1. PDAC‐PDX response to cremophor EL‐paclitaxel (crem‐PTX) and combined with Gemcitabine (Gem). Fig. S2. Gemcitabine double dose did not improve response to therapy. Fig. S3. Microenvironment contribution to HuPa11 responsiveness to chemotherapy. Fig. S4. Quantification of stroma abundance in pancreatic ductal adenocarcinoma patient‐derived xenografts (PDAC‐PDXs). Fig. S5. PDAC‐PDX responsiveness is not associated with a different pharmacokinetic profile or intratumor distribution of paclitaxel (PTX). Fig. S6. Clustering of PDAC‐PDXs based on expression of 24‐stroma gene sub‐signature and Moffitt‐activated stroma genes. [file MOL2-19-1075-s003.zip › MOL213816-sup-0002-FigS2.tif]

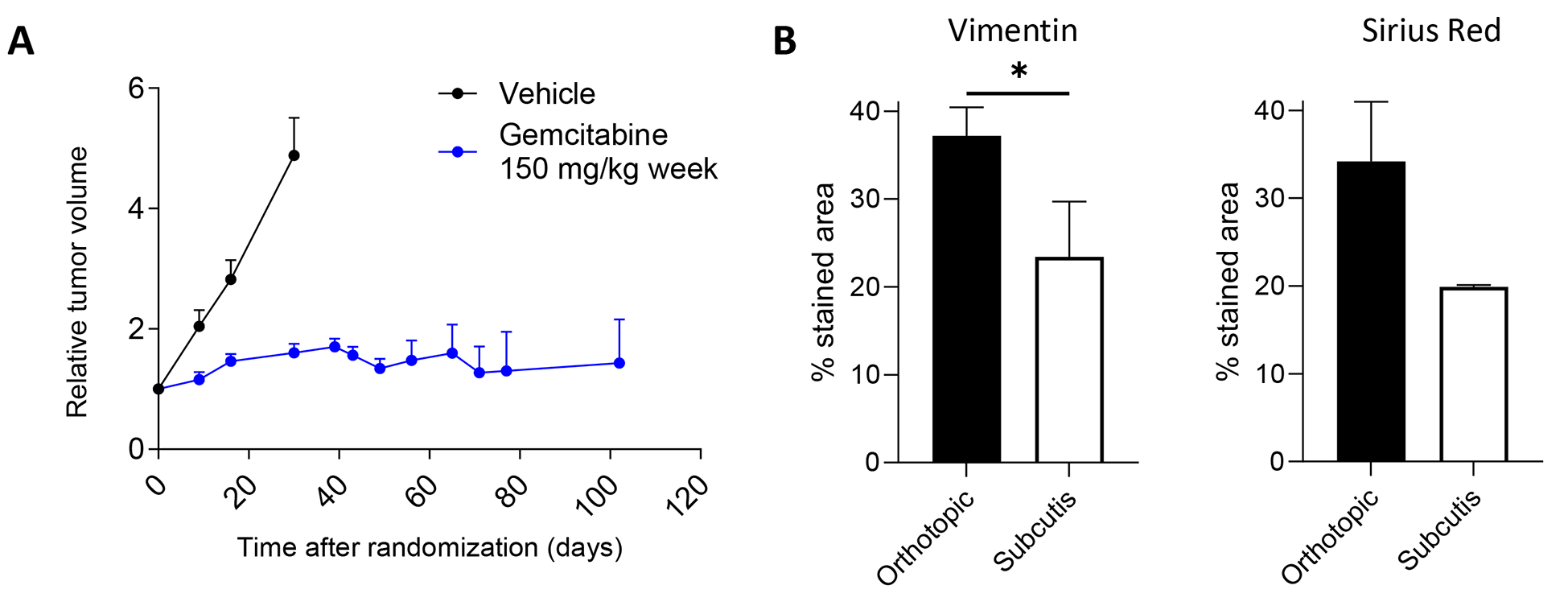

Supplement: Supplementary file 1 — Fig. S1. PDAC‐PDX response to cremophor EL‐paclitaxel (crem‐PTX) and combined with Gemcitabine (Gem). Fig. S2. Gemcitabine double dose did not improve response to therapy. Fig. S3. Microenvironment contribution to HuPa11 responsiveness to chemotherapy. Fig. S4. Quantification of stroma abundance in pancreatic ductal adenocarcinoma patient‐derived xenografts (PDAC‐PDXs). Fig. S5. PDAC‐PDX responsiveness is not associated with a different pharmacokinetic profile or intratumor distribution of paclitaxel (PTX). Fig. S6. Clustering of PDAC‐PDXs based on expression of 24‐stroma gene sub‐signature and Moffitt‐activated stroma genes. [file MOL2-19-1075-s003.zip › MOL213816-sup-0003-FigS3.tif]

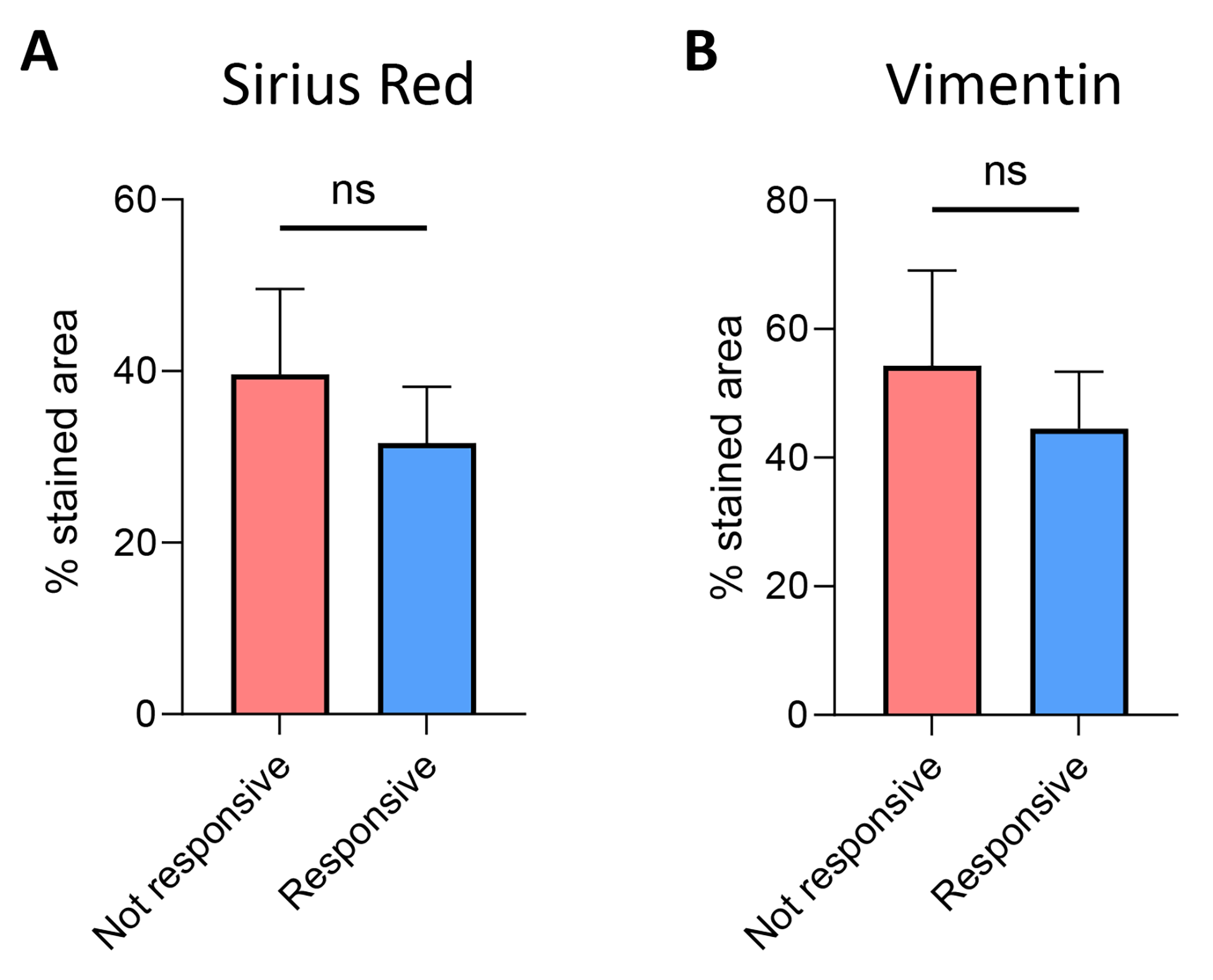

Supplement: Supplementary file 1 — Fig. S1. PDAC‐PDX response to cremophor EL‐paclitaxel (crem‐PTX) and combined with Gemcitabine (Gem). Fig. S2. Gemcitabine double dose did not improve response to therapy. Fig. S3. Microenvironment contribution to HuPa11 responsiveness to chemotherapy. Fig. S4. Quantification of stroma abundance in pancreatic ductal adenocarcinoma patient‐derived xenografts (PDAC‐PDXs). Fig. S5. PDAC‐PDX responsiveness is not associated with a different pharmacokinetic profile or intratumor distribution of paclitaxel (PTX). Fig. S6. Clustering of PDAC‐PDXs based on expression of 24‐stroma gene sub‐signature and Moffitt‐activated stroma genes. [file MOL2-19-1075-s003.zip › MOL213816-sup-0004_FigS4.tif]

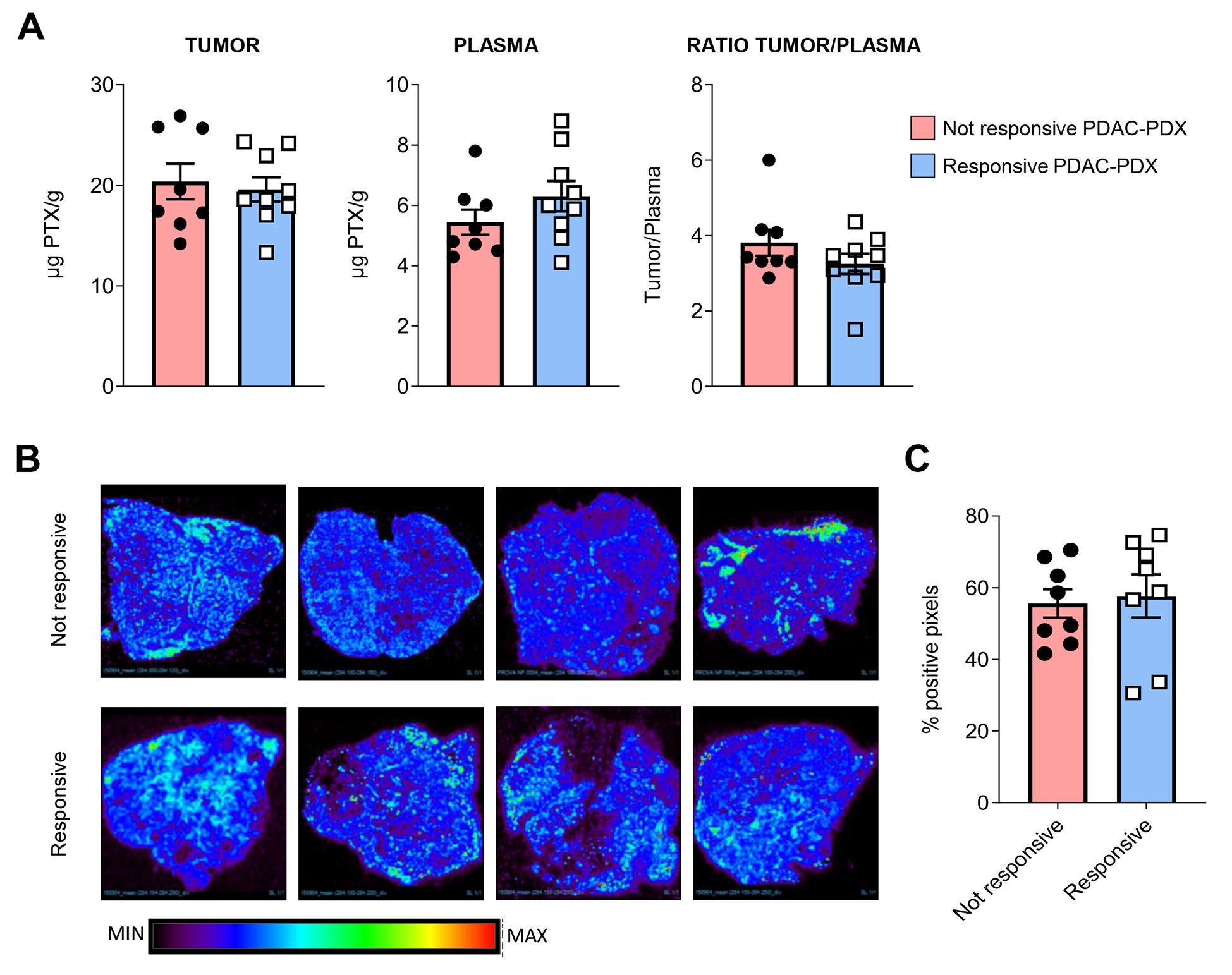

Supplement: Supplementary file 1 — Fig. S1. PDAC‐PDX response to cremophor EL‐paclitaxel (crem‐PTX) and combined with Gemcitabine (Gem). Fig. S2. Gemcitabine double dose did not improve response to therapy. Fig. S3. Microenvironment contribution to HuPa11 responsiveness to chemotherapy. Fig. S4. Quantification of stroma abundance in pancreatic ductal adenocarcinoma patient‐derived xenografts (PDAC‐PDXs). Fig. S5. PDAC‐PDX responsiveness is not associated with a different pharmacokinetic profile or intratumor distribution of paclitaxel (PTX). Fig. S6. Clustering of PDAC‐PDXs based on expression of 24‐stroma gene sub‐signature and Moffitt‐activated stroma genes. [file MOL2-19-1075-s003.zip › MOL213816-sup-0005-FigS5.tif]

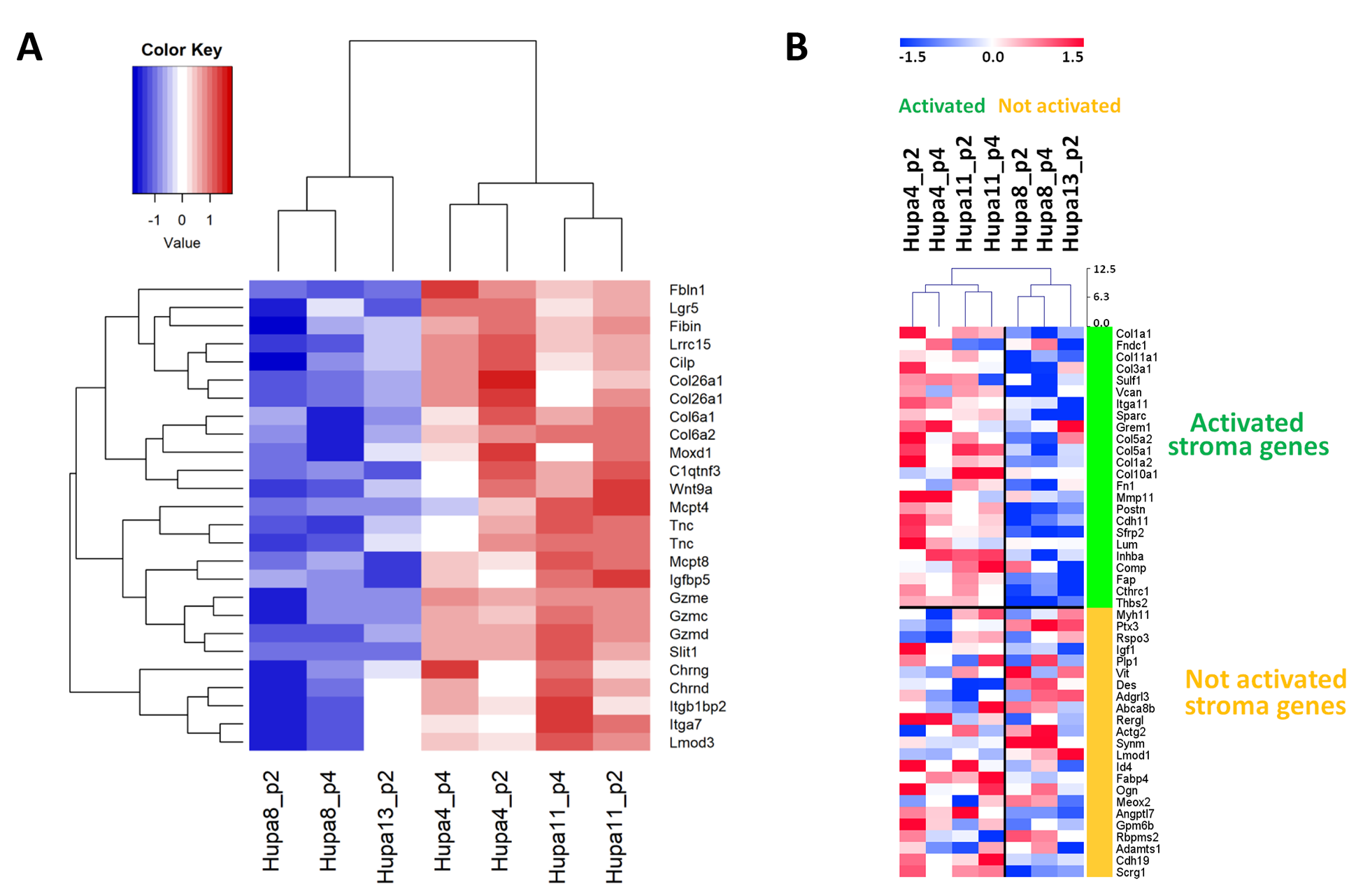

Supplement: Supplementary file 1 — Fig. S1. PDAC‐PDX response to cremophor EL‐paclitaxel (crem‐PTX) and combined with Gemcitabine (Gem). Fig. S2. Gemcitabine double dose did not improve response to therapy. Fig. S3. Microenvironment contribution to HuPa11 responsiveness to chemotherapy. Fig. S4. Quantification of stroma abundance in pancreatic ductal adenocarcinoma patient‐derived xenografts (PDAC‐PDXs). Fig. S5. PDAC‐PDX responsiveness is not associated with a different pharmacokinetic profile or intratumor distribution of paclitaxel (PTX). Fig. S6. Clustering of PDAC‐PDXs based on expression of 24‐stroma gene sub‐signature and Moffitt‐activated stroma genes. [file MOL2-19-1075-s003.zip › MOL213816-sup-0006-FigS6.tif]
