# Supplementary material for: Stroma gene signature predicts responsiveness to chemotherapy in pancreatic ductal adenocarcinoma patient‐derived xenograft models
Source: Mol Oncol. 2025 Feb 4;19(4):1075–91. doi: 10.1002/1878-0261.13816 (PMC11977644; doi:10.1002/1878-0261.13816)
Supplement: Supplementary file 2 — Table S1. Primers used for PCR amplification and sequencing and annealing temperature for each primer pair are indicated. [file MOL2-19-1075-s001.pdf]

Target	Forward primer (5'-3')	Reverse primer (5'-3')
***KRAS* exon 2**	CTTAAGCGTCGATGGAGGAG	AGAATGGTCCTGCACCAGTAA
***TP53* exon 5-6**	TTGCTTTATCTGTTCACTTGTGC	GCCACTGACAACCACCCTTA
***TP53* exon 7**	TGCTTGCCACAGGTCTCC	GGTCAGAGGCAAGCAGAGG
***TP53* exons 8-9**	CAAGGGTGGTTGGGAGTAGA	CCCCAATTGCAGGTAAAACA
***CDKN2A* exon 1**	TGGGTCCCAGTCTGCAGT	TAGCCTGGGCTAGAGACG
***CDKN2A* exon 2**	CTTGCCAACGCTGGCTCT	TGCAGACCCTCTACCCACCT
***CDKN2A* exon 3**	CCTGGCTCTGACCATTCTGT	TGGAAGCTCTCAGGGTACAAA
***CDKN2A* exon 4**	CGGTAGGGACGGCAAGAGA	GCCATTTGCTAGCAGTGTGA
***SMAD4* 1**	TCCTTGCAACGTTAGCTGTT	GTGAAGATCAGGCCACCTCC
***SMAD4* 2**	ATTGGATGGGAGGCTTCAGG	GCTGGGGTGCTGTATGTCTC
***SMAD4* 3**	CCATCCAGCATCCACCAAGT	AACAGGCCAGTAATGTCCGG
***SMAD4* 4**	CCAAAACGGCCATCTTCAGC	GAACAGCATCTCCAGGTGCA
***SMAD4* 5**	CCACGCGGTCTTTGTACAGA	TTAAGGGCCCCAACGGTAAA

Supplementary Table 1.
